# Comparison of phosphorylation and assembly of photosystem complexes and redox homeostasis in two wheat cultivars with different drought resistance

**DOI:** 10.1038/s41598-017-13145-1

**Published:** 2017-10-05

**Authors:** Yang-Er Chen, Jun-Mei Cui, Yan-Qiu Su, Chao-Ming Zhang, Jie Ma, Zhong-Wei Zhang, Ming Yuan, Wen-Juan Liu, Huai-Yu Zhang, Shu Yuan

**Affiliations:** 1 College of Life Sciences, Sichuan Agricultural University, Ya’an, 625014 China; 2 College of Life Science, Sichuan University, Chengdu, 610064 China; 3 College of Resources, Sichuan Agricultural University, Chengdu, 611130 China; 4 Center of Analysis and Testing, Sichuan Academy of Agricultural Sciences, Chengdu, 610066 China

## Abstract

Reversible phosphorylation of proteins and the assembly of thylakoid complexes are the important protective mechanism against environmental stresses in plants. This research was aimed to investigate the different responses of the antioxidant defense system and photosystem II (PSII) to osmotic stress between drought-resistant and drought-susceptible wheat cultivars. Results showed that the decrease in PSII photochemistry and six enzyme activities was observed in drought-susceptible wheat compared with drought-resistant wheat under osmotic stress. In addition, a lower accumulation of reactive oxygen species (ROS) and cell death were found in the resistant wheat compared with the susceptible wheat under osmotic stress. Western blot analysis revealed that osmotic stress led to a remarkable decline in the steady state level of D1 protein in drought-susceptible wheat. However, the CP29 protein was strongly phosphorylated in drought-resistant wheat compared with the susceptible wheat under osmotic stress. Our results also showed that drought-resistant wheat presented higher phosphorylated levels of the light-harvesting complex II (LHCII), D1, and D2 proteins and a more rapid dephosphorylated rate than drought-susceptible wheat under osmotic stress. Furthermore, the PSII-LHCII supercomplexes and LHCII trimers were more rapidly disassembled in drought-susceptible wheat than the drought-resistant wheat under osmotic stress. These findings provide that reversible phosphorylation of thylakoid membrane proteins and assembly of thylakoid membrane complexes play important roles in plant adaptation to environmental stresses.

## Introduction

Wheat (*Triticumae stivum* L.) is one of the most important food crops planted in the world and a staple food for 35–40% of the world population^1,2^. However, wheat plants often suffer from different environmental stresses under natural conditions. Water deficit caused by drought and osmotic stress is an important limitation for plants and affects severely changes in morphology, water status, gas exchange and chlorophyll content, which are related with the onset of protective mechanisms in plants^2–4^. Application of polyethylene glycol (PEG) in a solution causes osmotic stress, which results in changes in the water statue of the tissues and decrease in plant growth^5^. Plant drought tolerance is considered to be a quantitative trait manifesting complex phenotypic and genetic control^6,7^. To avoid or remove the harmful effects of drought stress, plants develop multiple strategies (morphological, physiological and biochemical). One of the most important adaptive mechanisms is the accumulation of osmotically active compounds, called osmotic adjustment (OA), which is considered an important feature of plant drought tolerance^8,9^.

Water stress is a complicated physiological and biochemical processes. During water stress, plants experience a lot of metabolic changes, including protein synthesis, gene expression, chlorophyll contents and production of reactive oxygen species (ROS)^10,11^. ROS are formed as a natural byproduct of the normal metabolism of oxygen and play an important role in plant signaling^12^. However, excessive amounts of ROS can cause lipid peroxidation^13^, damage chloroplast, inhibit photochemical reactions and decrease photosynthetic efficiency^14^. Osmotic stress invariably results in ROS generation. For surviving, plants adopt several mechanisms to counteract the adverse effects of stress-induced ROS. To scavenge ROS, as one of the defense mechanisms, plants develop enzymatic and non-enzymatic antioxidant defense systems^15^. Previous studies have demonstrated that water-stress-tolerant plants have low levels of lipid peroxidation together with higher antioxidant defense activities^16,17^.

PSII is a multi-subunit protein-complex embedded in the thylakoids of cyanobacteria, algae and plant cells. In higher plants, PSII plays an important role in the responses to various environmental stresses^11,18^. However, photosynthetic drought responses are complex and strongly depend on the plant development^19^. Pigment content, photochemical activity, phosphorylation of PSII proteins, thylakoid ultrastructure, and stomatal conductance are classically measured to evaluate the structural and functional changes in the photosynthetic apparatus of plants under environment stress^20–23^. In PSII, the occurrence of ROS usually caused the damage to thylakoid membranes. The report in algae showed that water stress led to a lower efficiency of photosynthetic electron transport^24^. Specific changes in fluorescence induction patterns also suggested that water stress hampered PSII activity^25^. Under moderate drought, a decrease in photosynthesis is generally considered to be the result of reduced availability of CO_2_ due to the stomatal closure^26^. Furthermore, previous studies had indicated that the steady-state levels of several PSII proteins remarkably decreased in barley exposed to osmoticstress^10,27^. In addition, many phosphorylated proteins, such as D1, D2, CP43, PsbH, TMP14, and CP29, had been identified under stressconditions^28,29^. In monocots, the phosphorylation of CP29 protein usually acted as an indicator under environmental stresses^30^. However, the differences in the levels of thylakoid proteins, the reversible phosphorylation of PSII proteins, and the assembly of thylakoid membrane complexes are remaining elusive between drought-resistant and drought-susceptible wheat cultivars.

At present, the topic of the antioxidant defense system and photosynthesis under biotic and abiotic stress has received much attention^31–34^. However, there was still few studies on the comprehensive comparisons of the photosynthetic characteristics and the antioxidant system between two wheat cultivars with different drought resistance under osmotic stress^35,36^. In this study, we compared the difference in chlorophyll fluorescence, ROS generation, activities of antioxidant enzymes, amounts of thylakoid proteins, phosphorylation of thylakoid membrane proteins and the structure of thylakoid between drought-susceptible and drought-resistant wheat cultivars exposed to osmotic stress. Here we expected to elucidate the regulatory role of the reversible phosphorylation of PSII proteins and thylakoid membrane complexes in the drought resistance of plants and provide a better understanding of stress resistant mechanisms in plants.

## Results

### Changes in symptoms, Chl content, RWC, total protein, soluble sugar, and free proline under osmotic stress

**Figure 1:**
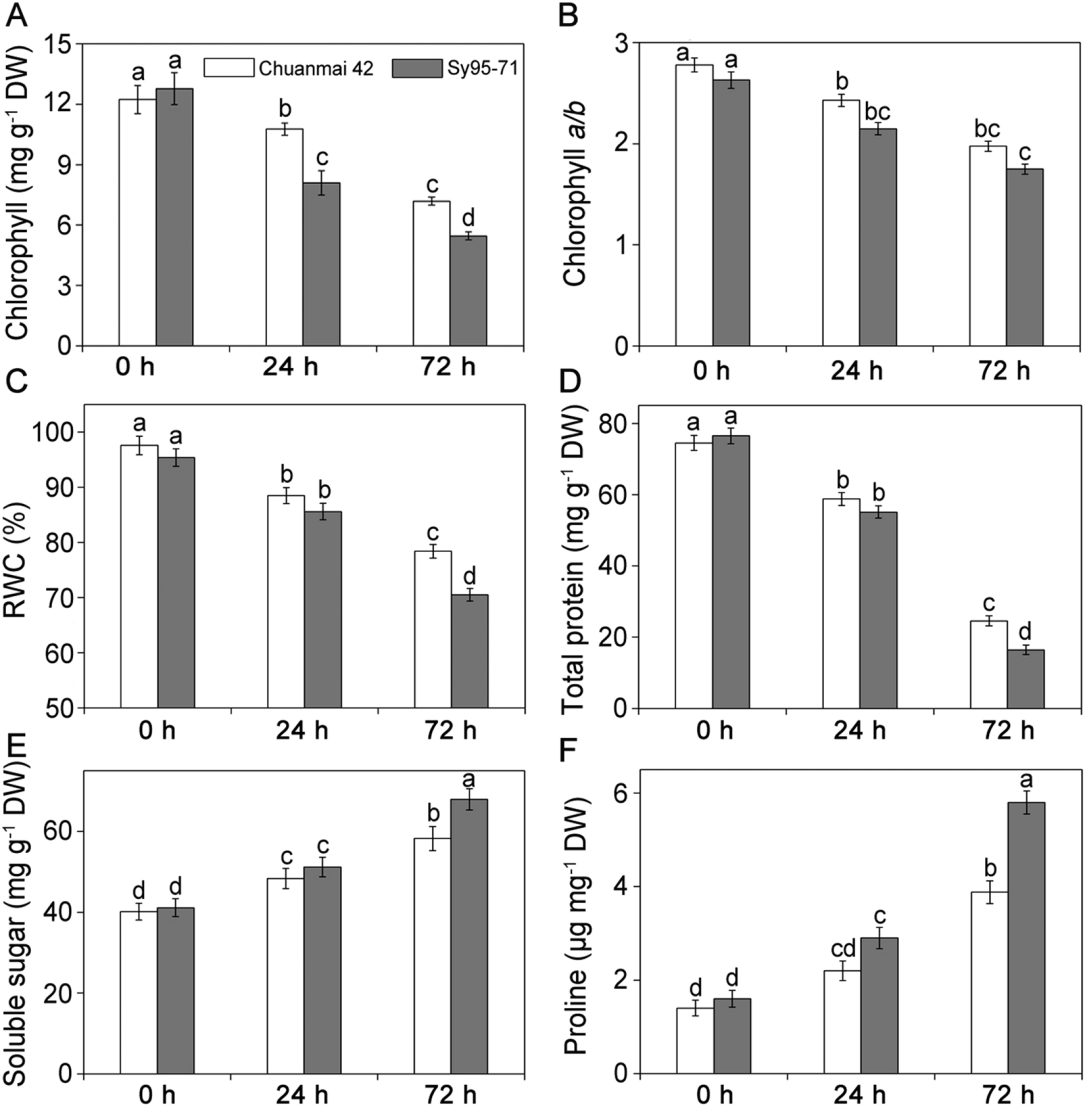
Effects of osmotic stress on chlorophyll content (**A**), Chl *a/b* ratio (**B**), RWC (**C**), total protein (**D**), soluable sugar (**E**), and proline (**F**) in Chuanmai42 and Sy95-71 wheat seedlings. Bars with different letters above the columns of figures indicate significant differences at *P* < 0.05 (Duncan’s multiple range test). 0–72 h represents osmotic stress for 0 h (control), 24 h, and 72 h.

Under the control and stressed conditions, the symptoms of two wheat cultivars were showed in Supplementary Fig. S1. Under non-stressful conditions, there were no obvious differences in symptoms between Chuanmai42 and Sy95–71. After 72 h of osmotic stress, Sy95–71 displayed a more obvious wilting compared to Chuanmai42, suggesting that Chuanmai42 was stronger in drought resistance than Sy95–71. No obvious differences in the content of Chl, RWC, total protein content, soluble sugar, and proline were observed between drought-resistant and drought-susceptible wheat under non-stressful conditions (Fig. 1). Compared with the control, Sy95-71 presented a remarkable decline in chlorophyll content under osmotic stress for 24 h in but not in Chuanmai42 (Fig. 1A). The ratio of Chl *a*/*b* significantly decreased in the two wheat cultivars compared to the control (Fig. 1B). Compared to the control plants, osmotic stress led to a remarkable decrease in RWC, from 97.1% to 88.3% and 78.4% after 24 h and 72 h of water deficit in Chuanmai42, and from 95.4% to 85.6% and 70.5% after 24 h and 72 h of osmotic stress in Sy95-71, respectively (Fig. 1C). In addition, RWC in Chuanmai42 was obviously higher than that of Sy95-71 under osmotic stress for 72 h. Compared to the control plants, the content of total protein obviously decreased by 59.0% and 88.9% under osmotic stress for 24 h and 72 h in Chuanmai42 and Sy95-71, respectively (Fig. 1D). Similar to RWC, the total protein content was significantly higher in Chuanmai42 than that of Sy95-71 after osmotic stress for 72 h.

Proline and soluble sugar, as the important osmotic regulators, usually accumulates under severe stress in plants^37^. Soluble sugar and proline content significantly increased in the leaves of Chuanmai42 and Sy95-71 after 24 h and 72 h of osmotic stress (Fig. 1E and F). Irrespective of experimental conditions, soluble sugar and proline levels were higher in Sy95-71 than that of Chuanmai42. After 72 h of osmotic stress, the content of soluble sugar and proline in Sy95-71 was 1.16 and 1.48 times of Chuanmai42, respectively.

### Osmotic stress induced lipid peroxidation and ROS accumulation

**Figure 2:**
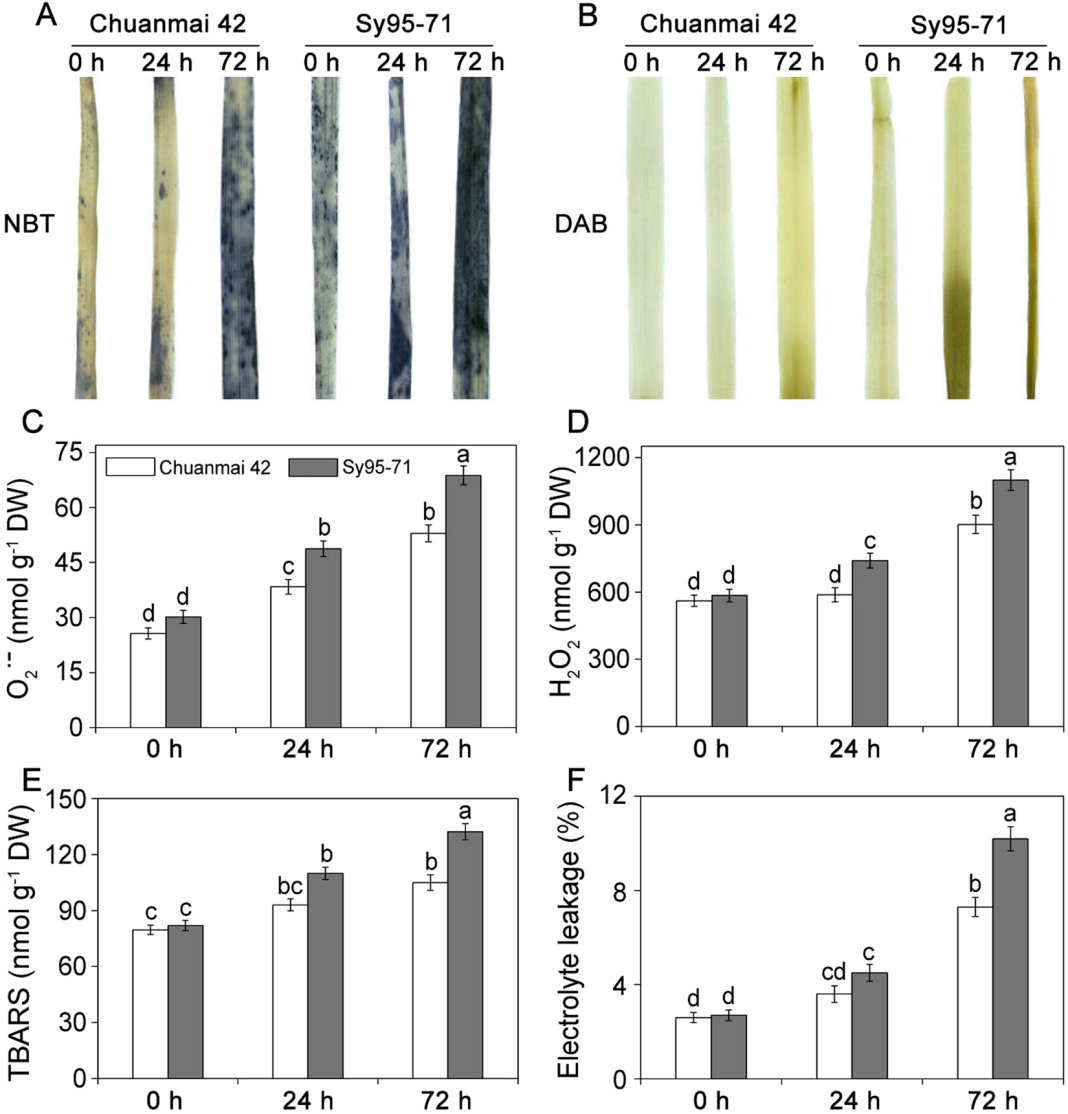
Effects of osmotic stress on ROS and lipid peroxidation in Chuanmai42 and Sy95-71 wheat seedlings. Histochemical analysis for superoxide anion radicals (O_2_^.−^) and hydrogen peroxide (H_2_O_2_) by nitro blue tetrazolium (NBT) (**A**) and 3,3-diaminobenzidine (DAB) (**B**) staining, respectively. Then, the content of O_2_^.−^ (**C**) and H_2_O_2_ (**D**), MDA (**E**), and electrolyte leakage (**F**) was measured. Bars with different letters above the columns of figures indicate significant differences at *P* < 0.05 (Duncan’s multiple range test). 0–72 h represents osmotic stress for 0 h (control), 24 h, and 72 h.

No obvious difference was observed between the controls of Chuanmai42 and Sy95-71 (Fig. 2A and B). However, both H_2_O_2_ and O_2_^.−^ levels were upregulated in the leaves of Chuanmai42 and Sy95-71 under osmotic stress, especially after 72 h, relative to those found in the control. Furthermore, the contents of H_2_O_2_ and O_2_^.−^ were more pronounced in drought-susceptible wheat (Sy95-71) than that of drought-resistant wheat (Chuanmai42).

To confirm these results, the contents of O_2_^.−^ and H_2_O_2_ in Chuanmai42 and Sy95-71 were quantified further under osmotic stress. Under osmotic stress for 24 h, the level of O_2_^.−^ and H_2_O_2_ in Chuanmai42 increased slightly relative to the control (Fig. 2C and D). However, the contents of O_2_^.−^ and H_2_O_2_ in Sy95-71 were obviously higher than that of Chuanmai42 under osmotic stress (*P* < 0.05). The level of O_2_^.−^ and H_2_O_2_ accumulation in Sy95-71 increased significantly by 127.5% and 96.6% under osmotic stress for 72 h, respectively. Moreover, the degree of oxidative damages in wheat leaves exposed to osmotic stress was examined by quantifying the levels of MDA and Electrolyte leakage (EL). As shown in Fig. 2E and F, 24 h of osmotic stress slightly increased the concentration of MDA and EL compared to the control. After 72 h of osmotic stress, the MDA content significantly increased by 31.9% and 60.9% compared to that of the respective controls in Chuanmai42 and Sy95-71, respectively. Compared with the respective control plants, the EL markedly increased by 180.7% and 277.8% in Chuanmai42 and Sy95-71 after 72 h of osmotic stress, respectively. The content of MDA and EL was significantly higher in Sy95-71 than that observed in Chuanmai42.

### Enzymatic and non-enzymatic antioxidant activities were different between the two wheat cultivars under osmotic stress

**Figure 3:**
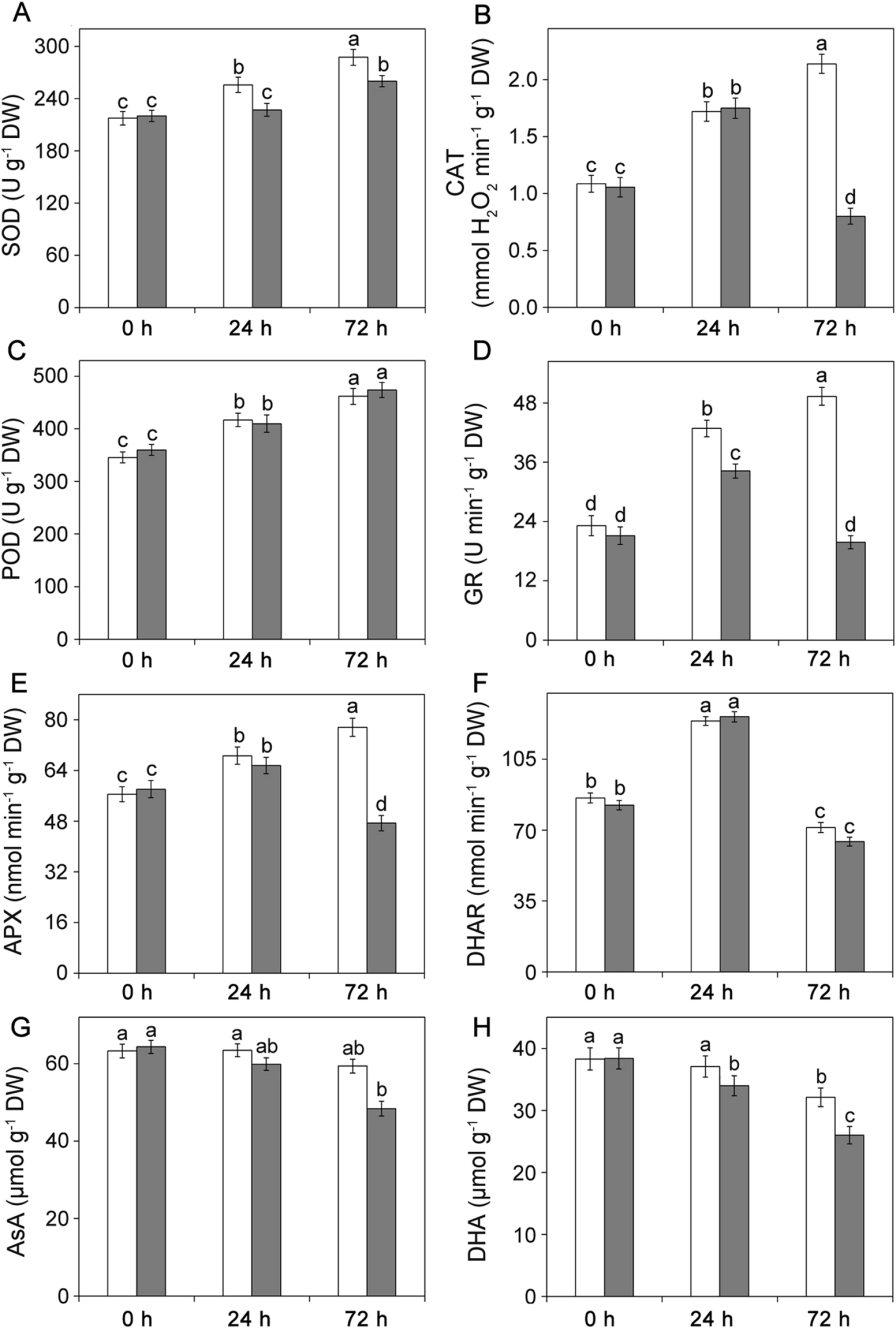
Effects of osmotic stress on the specific activities of SOD (**A**), CAT (**B**), POD (**C**), GR (**D**), APX (**E**), DHAR (**F**), AsA (**G**) and DHA (**H**) in drought-susceptible and drought-resistant wheat cultivars. Bars with different letters above the columns of figures indicate significant differences at *P* < 0.05 (Duncan’s multiple range test). 0–72 h represents osmotic stress for 0 h (control), 24 h, and 72 h.

Changes in the activities of the non-enzymatic antioxidants and antioxidant enzymes in two wheat cultivars exposed to osmotic stress were shown in Fig. 3. When compared with the control plants, Chuanmai42 showed the marked increase in the activities of CAT, SOD, POD, GR, and APX under osmotic stress (Fig. 3A,B,C,D and E). However, the activities of DHAR significantly decreased under osmotic stress for 72 h in Chuanmai42 and Sy95-71 (Fig. 3F). Although Sy95-71 also showed increases in the activities of some antioxidant enzymes under osmotic stress relative to the control plants, more obvious increases were observed in Chuanmai42, especially under osmotic stress for 72 h. In addition, CAT, APX, and DHAR activities obviously decreased after 72 h of osmotic stress in Sy95-71 relative to the control plants (Fig. 3B,E and F). AsA and DHA are important antioxidant molecules in plants. The decrease in the contents of AsA and DHA occurred under osmotic stress for 72 h in Chuanmai42 compared to the control (Fig. 3G and H). However, the contents of AsA and DHA in Sy95-71 significantly increased after 24 h of osmotic stress compared with the control plants. In addition, Sy95-71 presented the lower contents of ASA and DHA under osmotic stress compared with Chuanmai42 (Fig. 3G and H).

### Effects of *in-vitro* AsA treatments on Chl, RWC, chlorophyll fluorescence and lipid peroxidation under osmotic stress

To test whether exogenous AsA was absorbed by seedlings, the concentration of AsA was measured under non-stressed condition. Compared to the control, AsA application led to a obvious increase in the content of AsA in seedlings (Supplementary Fig. S2A), indicating that exogenous AsA could be taken up effectively by wheat seedlings. Furthermore, we also examined the effects of AsA application on ROS accumulation under non stressful conditions. The results showed that the application of exogenous AsA resulted in a slight increase in the levels of O_2_^.−^ and H_2_O_2_ compared with the control plants in both wheat cultivars (Supplementary Fig. S2B and C).

To further investigate the relationship between the antioxidative system and drought tolerance of Chuanmai42 and Sy95-71, Chl, RWC, Fv/Fm, NPQ, MDA, and EL were measured in the two wheat cultivars exposed to *in-vitro* AsA application under osmotic stress. As shown in Supplementary Fig. S3, AsA application resulted in slight increases in Fv/Fm, Chl contents, and RWC under non-stressed condition compared to the control (Supplementary Fig. S3A,C and D). In contrast, the declines in NPQ and MDA content and EL were found for the AsA treatment (Supplementary Fig. S3B,E and F). While the PEG-induced osmotic stress still resulted in declines in Fv/Fm, Chl content and RWC and increases in NPQ, MDA and EL in Chuanmai42 and Sy95-71. After 72 h of osmotic stress, Chl content represented 15.4% and 12.9% recovery in Chuanmai42 and Sy95-71 exposed to AsA application relative to 72 h of PEG-6000 treatment without AsA application, respectively. In addition, compared to 72 h of osmotic stress in the absence of exogenous AsA, the concentration of MDA decreased by 20.9% and 23.7% in Chuanmai42 and Sy95-71 exposed to AsA application under osmotic stress for 72 h, respectively. These results indicated that exogenous AsA application improved the drought tolerance both in two wheat cultivars. But the increase in tolerance showed no significant difference between Chuanmai42 and Sy95-71 (Supplementary Fig. S3). The data imply that the differences of stress tolerance between two wheat cultivars may be attributed to their different antioxidant abilities.

### Changes in characterization of PSII under osmotic stress

**Figure 4:**
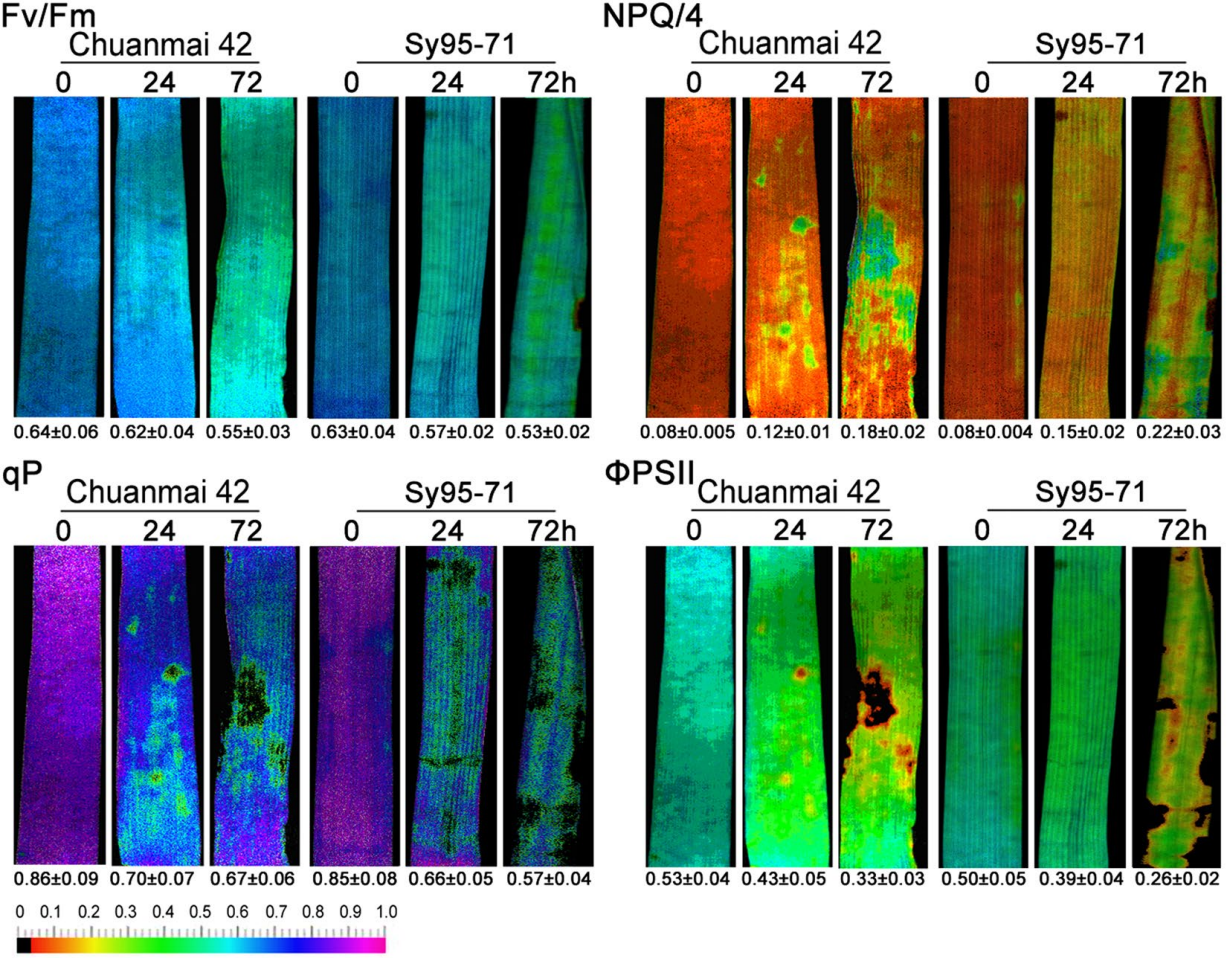
Effects of osmotic stress on chlorophyll fluorescence parameters (the PSII maximum quantum yield, Fv/Fm; the non-photochemical quenching coefficient, NPQ/4; the photochemical quenching coefficient, qP and the effective quantum yield of PSII photochemistry, ΦPSII) in Chuanmai42 and Sy95-71. Values below the individual images present quantitative means ± SD (*n* = 3). 0–72 h represents osmotic stress for 0 h (control), 24 h, and 72 h.

To analyze the effects of osmotic stress on PSII photochemistry in two wheat cultivars, chlorophyll fluorescence images and value were detected by using a modulated imaging fluorometer. The color and value of Fv/Fm, qP, ΦPSII, and NPQ in the control plants of Chuanmai42 and Sy95-71 showed no significant differences (Fig. 4). Compared to Chuanmai42, Sy95-71 presented no obvious difference in Fv/Fm and slight decreases in ΦPSII and qP under osmotic stress for 24 h (Fig. 4). However, osmotic stress for 72 h resulted in more significant decreases in ΦPSII and qP and an increase in NPQ in Sy95-71 compared to Chuanmai42, indicating that Chuanmai42 has higher photochemical efficiency than Sy95-71.

**Figure 5:**
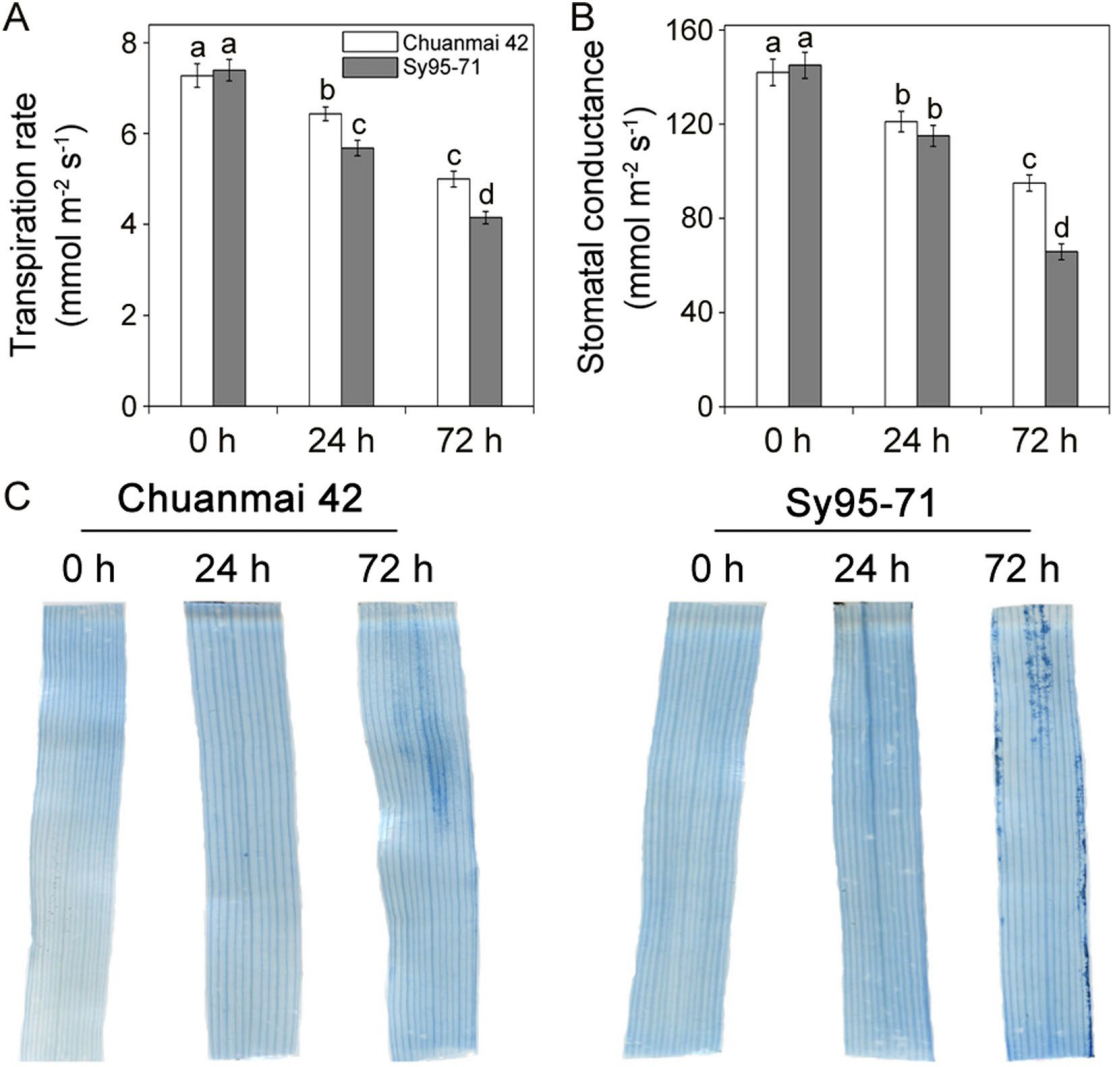
Effects of osmotic stress on transpiration rate, stomatal conductance, and cell death in drought-susceptible and drought-resistant wheat cultivars. The second leaves of Chuanmai42 and Sy95-71 exposed to control and PEG-6000-stressed conditions were stained with trypan blue. Bars with different letters above the columns of figures indicate significant differences at *P* < 0.05 (Duncan’s multiple range test). 0–72 h represents osmotic stress for 0 h (control), 24 h, and 72 h.

In addition, the transpiration rate and stomatal conductance were further compared between drought-susceptible and drought-resistant wheat under osmotic stress (Fig. 5A and B). Compared to Sy95-71, Chuanmai42 displayed higher transpiration rate under osmotic stress for 24 h and 72 h. However, more significant decreases in stomatal conductance were observed in Sy95-71 compared to Chuanmai42 after 72 h of osmotic stress.

### Effect of osmotic stress on cell death in two wheat cultivars

To investigate whether severe oxidative damage was involved in cell death in Sy95-71, the leaves of two wheat cultivars exposed to osmotic stress were stained with trypan blue. The results obtained from trypan-blue staining revealed that the leaves of Chuanmai42 and Sy95-71 under control condition and osmotic stress for 24 h showed no intensely stained areas (Fig. 5C). However, the obvious cell death was observed in Sy95-71 exposed to osmotic stress for 72 h but not in Chuanmai42 (Fig. 5C), indicating that Sy95-71 suffered more severe cell damage than Chuanmai42 under osmotic stress.

### Effect of osmotic stress on the thylakoid protein contents and phosphorylation levels

**Figure 6:**
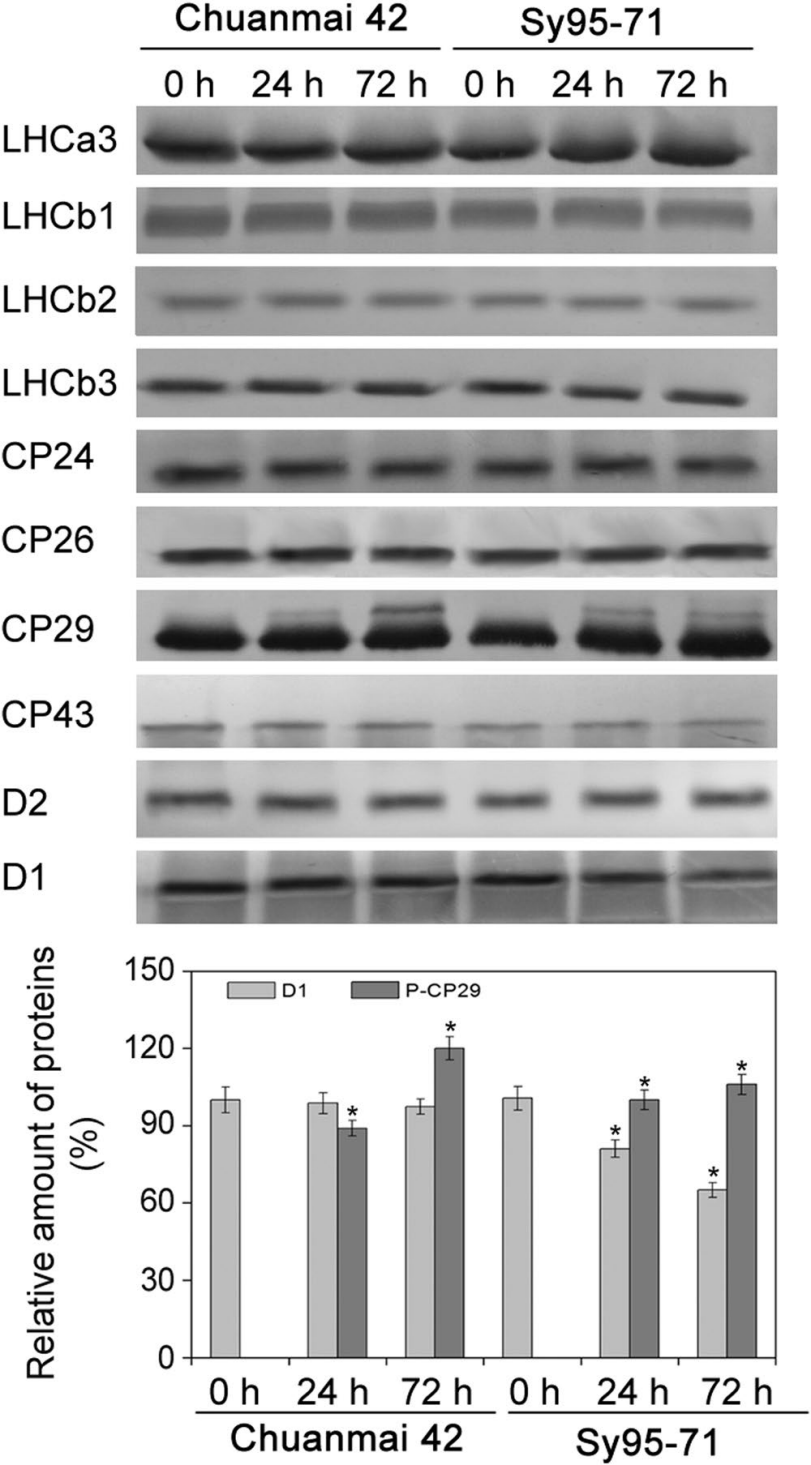
Accumulation of PSII proteins from Chuanmai42 and Sy95-71 under osmotic stress. Immunoblotting of thylakoids was done using specific antisera against Lhca3, Lhcb1, Lhcb2, Lhcb3, CP24, CP26, CP29, CP43, D2, and D1 (**A**). Then, quantitative analyses for phosphorylated-CP29 and D1 proteins are shown (**B**). Data are normalized relative to the content of control (100%). Asterisk indicates significant differences at *P* < 0.05 level (*n* = 4). Loading was according to equal amount of total chlorophyll (1 μg of Chl). 0-72 h represents osmotic stress for 0 h (control), 24 h, and 72 h.

**Figure 7:**
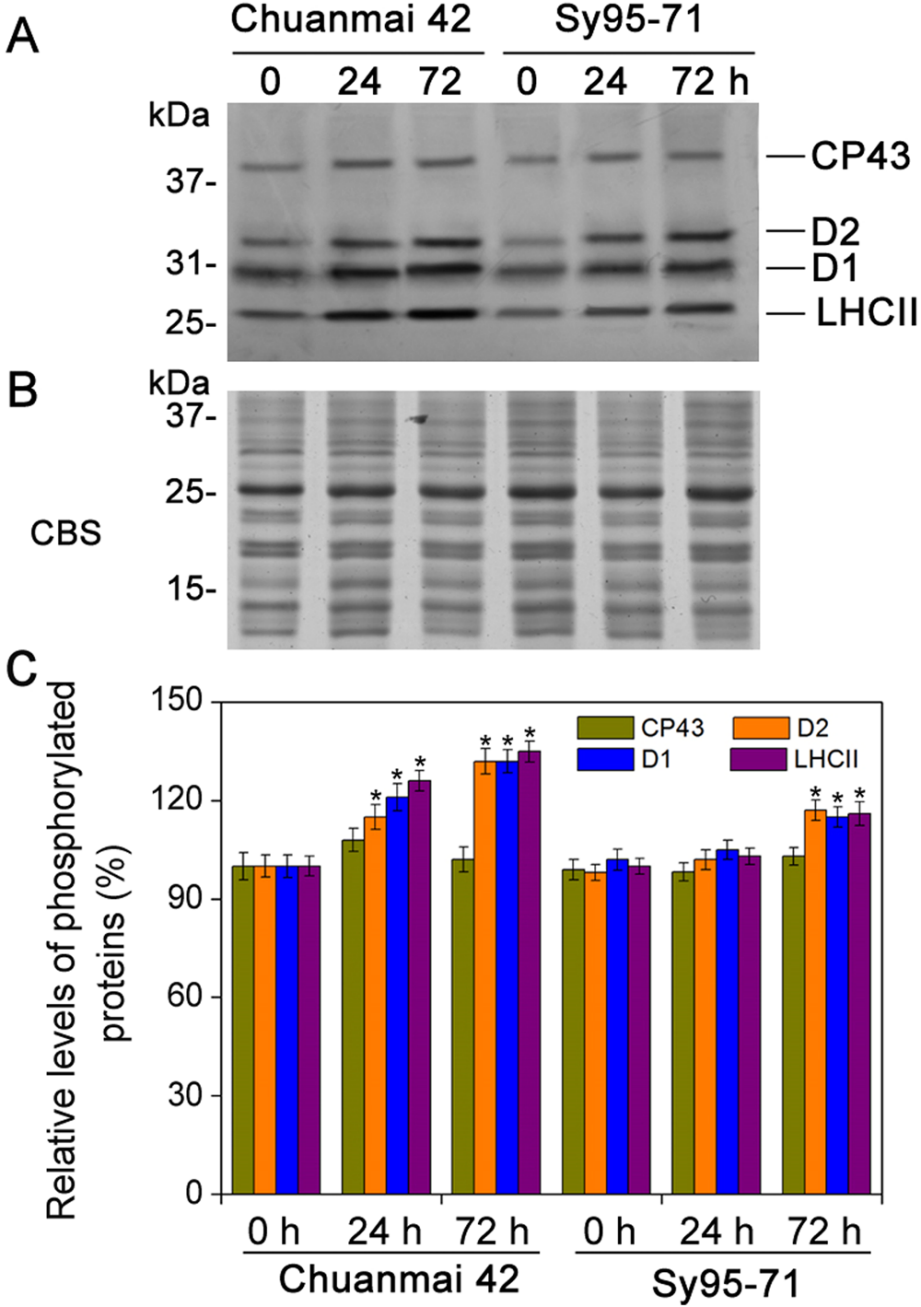
Analyses of thylakoid protein phosphorylation under osmotic stress in Chuanmai42 and Sy95-71. Immunoblotting of thylakoid membrane proteins was done with anti-phosphothreonine antibodies (**A**). Two microgram of chlorophyll (2 μg of Chl) was loaded into each electrophoretic lane. (**B**) Coomassie blue staining (CBS) after SDS-PAGE is showed. (**C**) Quantitative analyses of thylakoid protein phosphorylation under osmotic stress. Relative amount are showed with respect to the content of 0 h (100%). Significant differences are presented with the asterisk at *P* < 0.05 level (Duncan’s multiplication range test). Data present the means ± SD (*n* = 4). 0–72 h represents osmotic stress for 0 h (control), 24 h, and 72 h.

To observe the effects of osmotic stress on thylakoid proteins between drought-susceptible and drought-resistant wheat, immunoblotting of thylakoid polypetide composition was performed in wheat. Almost no detectable changes in the amount of almost all the analyzed thylakoid membrane proteins were found under osmotic stress between Chuanmai42 and Sy95-71 (Fig. 6). Only the level of D1 protein under osmotic stress was significantly reduced compared to the control in Sy95-71. Although there was no significant difference in the content of the CP29 protein in two wheat cultivars under osmotic stress, CP29 was phosphorylated under osmotic stress relative to the control plants in Chuanmai42 and Sy95-71 (Fig. 6). However, compared with Sy95-71, osmotic stress for 72 h resulted in a more obvious phosphorylation of CP29 in Chuanmai42. We further investigated the phosphorylation pattern of PSII proteins by the immunoblotting. We found that the phosphorylated level of CP43 protein did not display remarkable change under osmotic stress, while osmotic stress resulted in an increase in the phosphorylation level of D1, D2, and LHCII in Chuanmai42 and Sy95-71compared with the control plants (Fig. 7A). In addition, higher levels of phosphorylated D1, D2, and LHCII were observed in Chuanmai42 than those of Sy95-71 under osmotic stress (Fig. 7).

### Effect of osmotic stress on dephosphorylation of the thylakoid proteins *in vivo*

**Figure 8:**
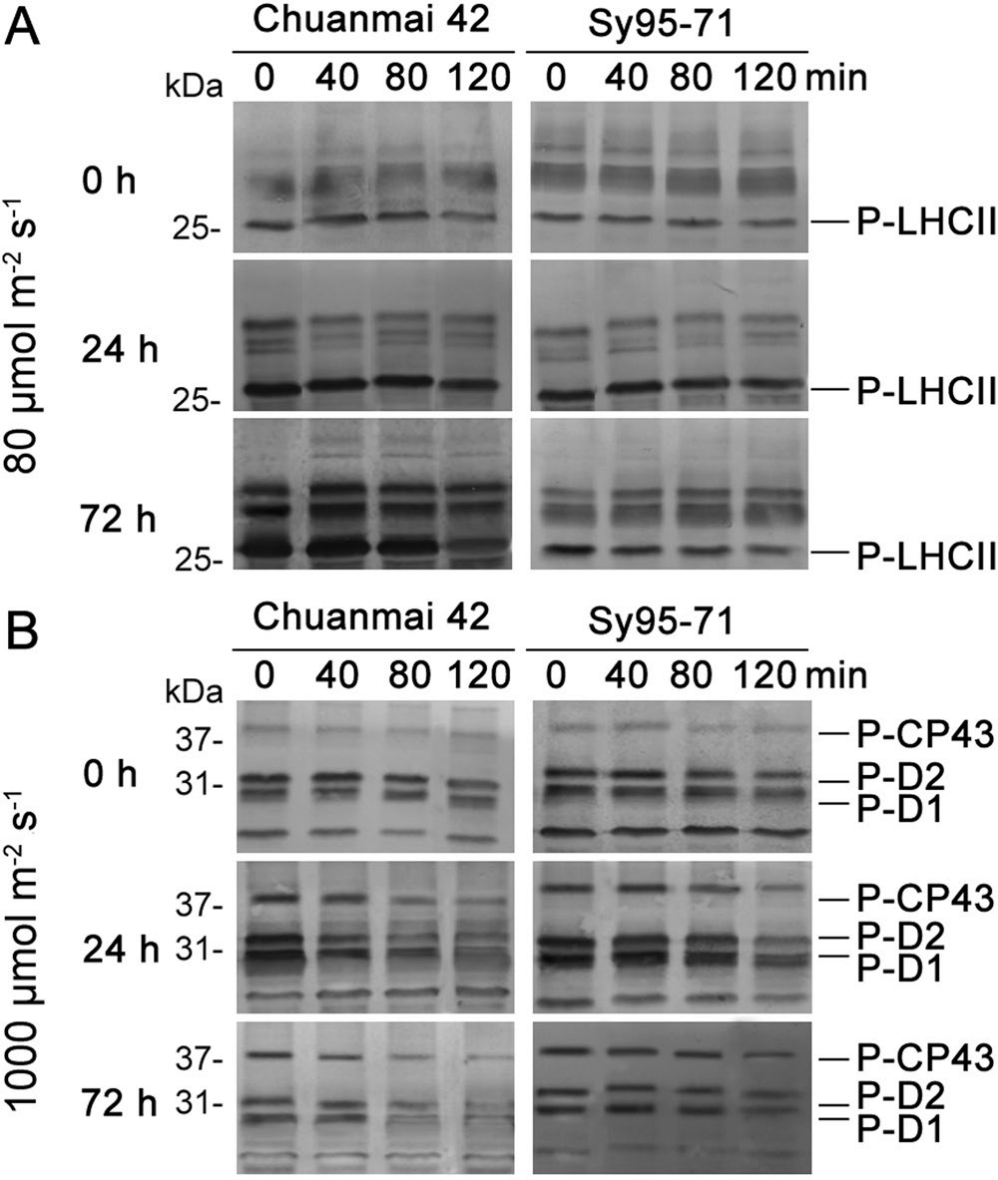
Dephosphorylation of PSII proteins from Chuanmai42 and Sy95-71 *in vivo* under osmotic stress. Wheat seedlings were illuminated under 80 μmol photons m^−2^ s^−1^ to induce maximal LHCII phosphorylation (**A**) or at 1000 μmol photons m^−2^ s^−1^ to phosphorylate PSII reaction center proteins (**B**) for 60 min at 25 °C, and subsequently transferred to darkness and incubated at 25 °C. Dephosphorylation was terminated at the indicated time points by freezing the leaves in liquid nitrogen. Thylakoid membranes were isolated and the extent of protein phosphorylation was determined using a phosphothreonine antibody. 0–72 h represents osmotic stress for 0 h (control), 24 h, and 72 h.

To investigate the physiological relevance of osmotic stress induced specific dephosphorylation of PSII proteins between Chuanmai42 and Sy95-71, the experiment *in vivo* was performed in the leaves during 120 min incubation in darkness at 25 °C as judged by analysis with the phosphothreonine antibody. We found that dephosphorylated behavior of CP43, D2, D1, and HLCII proteins was similar (Fig. 8). In the unstressed condition (0 h), these proteins dephosphorylated slowly (half-times were less than 120 min). However, osmotic stress accelerated the dephosphorylated rates of all PSII proteins. Only 72 h of osmotic stress significantly increased dephosphorylation of LHCII proteins, especially in Chuanmai42 (Fig. 8A). But CP43, D2, and D1 proteins obviously dephosphorylated after 24 h of osmotic stress (Fig. 8B). In addition, we also found that dephosphorylated rates of all these PSII proteins under osmotic stress were higher in Chuanmai42 than that observed in Sy95-71.

### Alterations in thylakoid membranecomplexes and the thylakoid ultrastructure of wheat plants under osmotic stress

**Figure 9:**
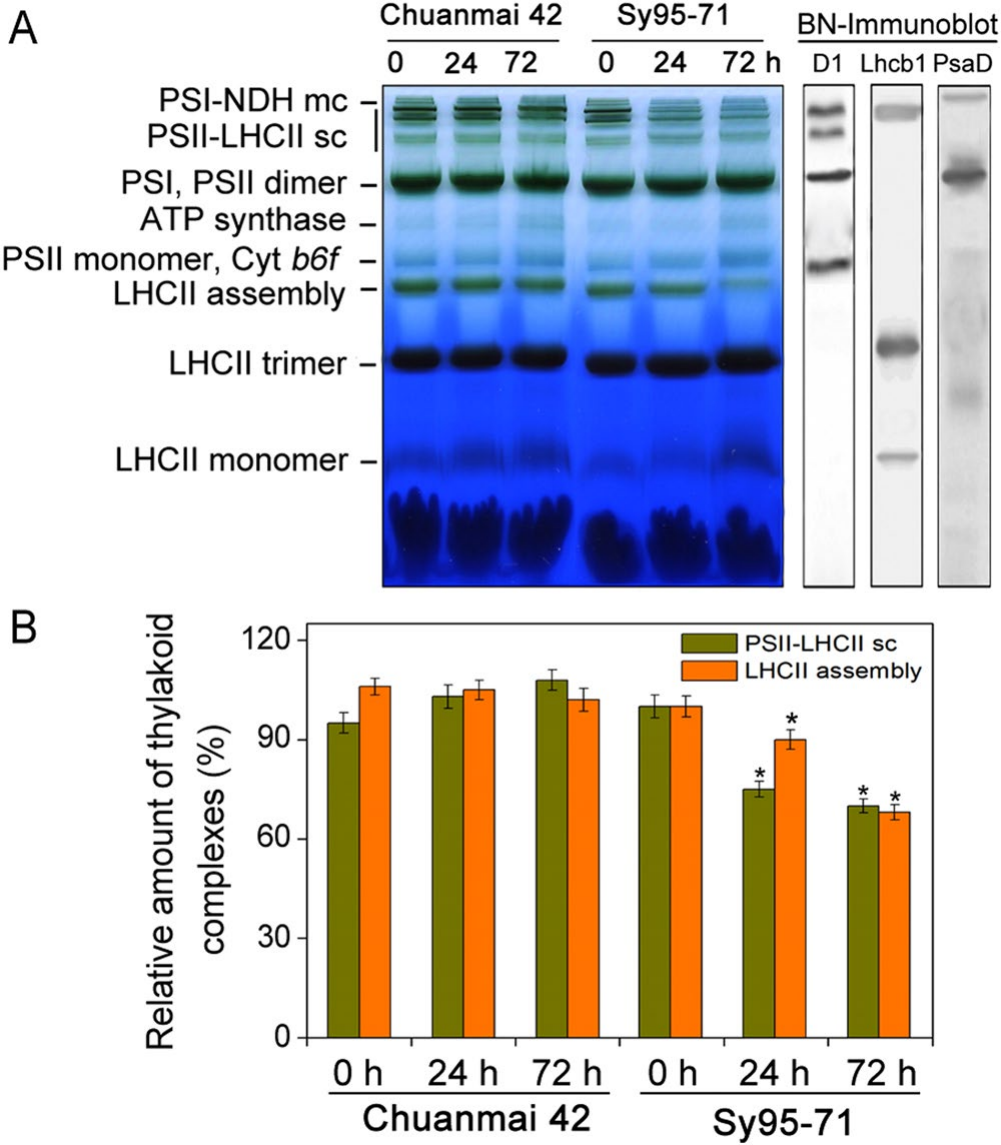
BN-PAGE analyses of thylakoid membrane protein complexes from Chuanmai42 and Sy95-71 under osmotic stress. Separation was achieved by BN-PAGE using 5-12.5% acrylamide. Thylakoid membranes (20 μg of Chl) solubilized using 1% (w/v) DM were subjected to BN-PAGE (**A**). PS, photosystem; NDH, NAD(P)H dehydrogenase; mc, megacomplex; sc, supercomplex; LHC, light-harvesting complex; Cyt *b*_6_ */f*, cytochrome *b*_6_ */f*. The bands of BN-PAGE were confirmed by immunoblotting with D1, Lhcb1, and PsaD specific antibodies (on the right). The control line of the BN gel was selected for immunodetection. (C) Quantitative analyses of LCHII assemblies and the PSII-LHCII supercomplexes under osmotic stress. Relative intensities of bands with respect to the content of 0 h (100%) are presented. Significant differences are presented with the asterisk at *P* < 0.05 level (Duncan’s multiplication range test). Values show the means ± SD (*n* = 4). 0–72 h represents osmotic stress for 0 h (control), 24 h, and 72 h.

To compare changes in the organization of thylakoid complexes between drought-resistant and drought-susceptible wheat under osmotic stress, thylakoid membrane complexes were separated by the BN-PAGE (Fig. 9). Relative to the control plants, a significant decrease in the amount of the PSII-LHCII supercomplexes was found under osmotic stress for 24 h in Sy95-71, but not in Chuanmai42. Similarly, 72 h of osmotic stress resulted in a marked reduction in the amount of LHCII assemblies. However, compared to the control plants, thylakoid membrane complexes showed no observed changes under osmotic stress in Chuanmai42.

**Figure 10:**
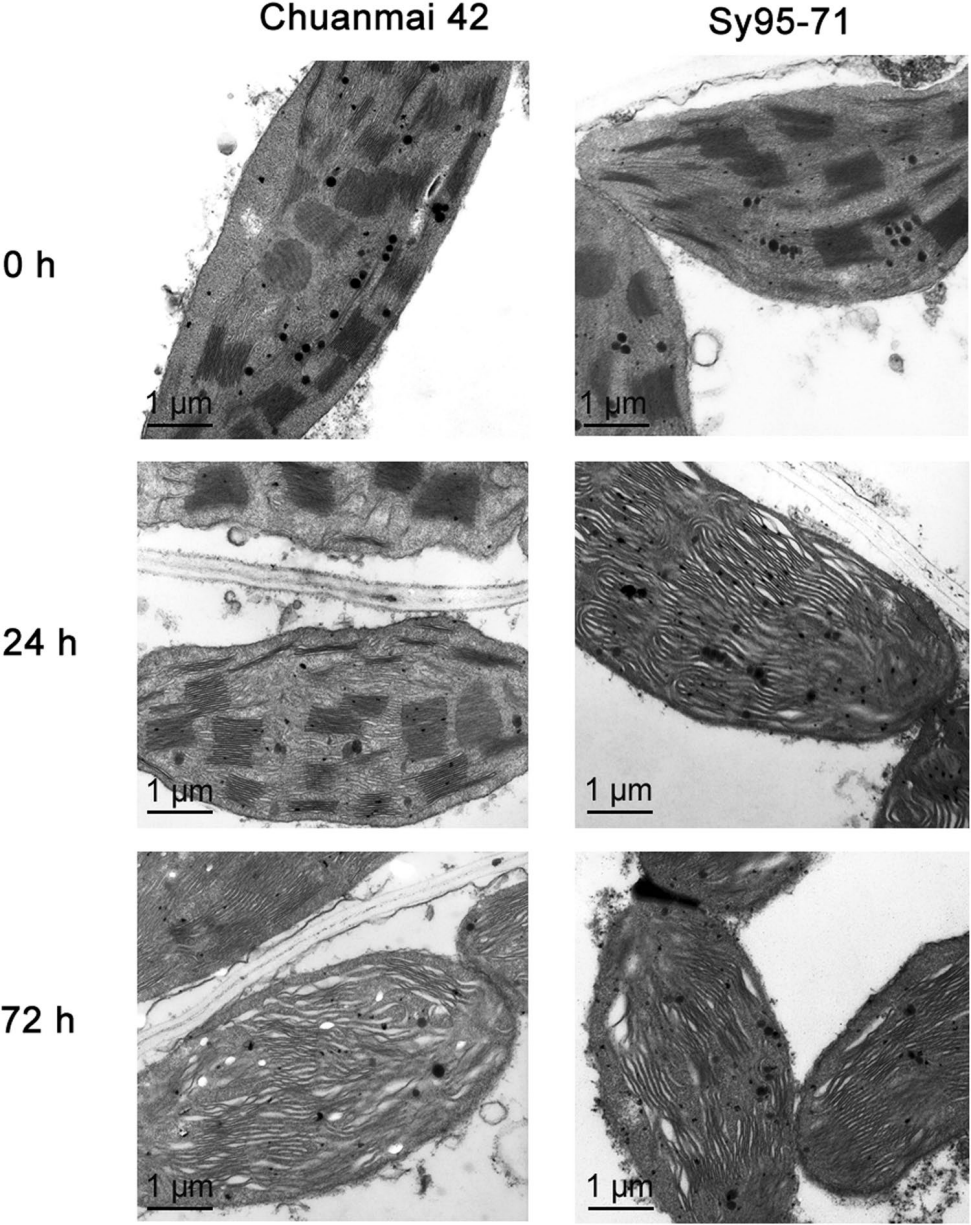
Transmission electron microscope analysis of chloroplasts from Chuanmai42 and Sy95-71 under osmotic stress. 0–72 h represents osmotic stress for 0 h (control), 24 h, and 72 h.

To further compare changes in thylakoid structures of Chuanmai42 and Sy95-71 under osmotic stress, the chloroplast structure was observed with a transmission electron microscopy. The stacking of the grana in Sy95-71 was obviously reduced under the stress conditions and the thylakoid became fibrous as compared to the control plants (Fig. 10). However, compared to the control, 24 h of osmotic stress did not result in remarkably changes in the thylakoid structure of Chuanmai42. In contrast, the significant destacking of the thylakoid membrane was observed under osmotic stress for 72 h in Chuanmai42.

## Discussion

It is well known that water stress is one of the most important environmental stresses and a major limitation for plant growth, development and yield^33,38^. Many studies have showed that osmotic stress affected gas exchange parameters, chlorophyll content, lipid peroxidation, the activities of antioxidant enzymes, PSII photochemistry, and the structure of thylakoid^11,33,38–40^. Although photosystem I (PSI) may be damaged by photoinhibition under environmental stresses, PSI is considered to be much resistant to photoinhibition and oxidative damages^21,41^. Therefore, we focus on PSII changes in the two wheat cultivars. In the present study, the differential responses of PSII and ROS to osmotic stress between drought-susceptible and drought-resistant wheat cultivars were compared.

It has been known that osmotic stress often leads to the decrease in total chlorophyll content and substantial damages to photosynthetic pigments^11,42^. In consistent with these previous studies, we found that the total Chl content of both wheat cultivars significantly decreased under osmotic stress, especially in drought-susceptible wheat, implying that drought-resistant wheat has a more effective protective system against damages to pigments caused by environmental stresses^31^. A previous study indicated that drought-resistant wheat had the more efficient protection to tissue water status compared with susceptible genotypes^43^. Our results are in consistence with the observation. In addition, we found that drought-resistant wheat had lower levels of soluble sugar and proline, which are the two most important organic solutes in higher plants under stress conditions^44^. It is well known that soluble sugar and free porline increase in response to drought in plants^44^, which was also confirmed here.

It has become apparent that ROS play a dual role in plants, on the one hand as important signal transduction molecules, and on the other hand as toxic by-products of aerobic metabolism that accumulate in cells under different environmental stresses^12^. This study showed that osmotic stress significantly induced cell death and ROS generation in drought-susceptible wheat genotypes, especially under osmotic stress for 72 h, which indicated that drought-susceptible wheat suffered more severe oxidative stress under osmotic stress compared with drought-resistant wheat. The high content of O_2_^.−^ and H_2_O_2_ in drought-susceptible wheat under osmotic stress could be evaluated by the levels of the electrolyte leakage and MDA^45^, which is common and important index for evaluating the redox status of wheat. In our experiment, we showed that lipid peroxidation was more severe in drought-susceptible wheat, which is consistent with the ROS accumulation level. This further implied that drought-resistant wheat has a different physiological adaptive mechanism to regulate its redox status from drought-susceptible wheat.

In order to remove the excessive ROS species and maintain redox homeostasis under abiotic stress conditions, a complex array of enzymatic and non-enzymatic antioxidant defense systems is engaged in plants^15^. The coordinated effort of antioxidant compounds and antioxidant enzymes contributes to oxidative stress adaptation. Several reports have showed that the activities of antioxidant enzymes increase in different plants under osmotic stress^46,47^. Here, we found that the activities of SOD, CAT, GR, and APX markedly increased in drought-resistant wheat compared with drought-susceptible wheat under osmotic stress, indicating that drought-resistant wheat has more effective antioxidant defense systems in the scavenging of ROS. Furthermore, AsA is a soluble compound and ubiquitous in photosynthetic organisms, acting as important antioxidant in plants^48^. A previous report stated that the decrease in AsA content and AsA-GSH cycle enzymes induces oxidative damages during severe water stress^43^. Lascano *et al*. reported that higher AsA content and induction of AsA-GSH cycle enzymes alleviated the oxidative damage in the tolerant wheat cultivar under osmotic stress^49^. Our results found that drought-susceptible wheat had the lower level of AsA and DHA compared with drought-resistant wheat, suggesting that drought-resistant wheat was more effective in alleviating oxidative damages by promoting the AsA-GSH cycle under the stress conditions.

Chlorophyll fluorescence analysis has become one of the most powerful and useful, non-invasive techniques for the detection of environmental stresses in multiple plant species^18,50^. Some studies have indicated that severe drought stress resulted in the reduction in photochemical efficiency of PSII in different time and different extent^11,22^. In consistent with previous studies, our results showed that drought-susceptible wheat displayed a obvious decrease in photosynthetic capacity with respected to drought-resistant wheat, suggesting that drought-susceptible wheat suffered severe damages under osmotic stress. Under severe osmotic stress, the lower qP and ΦPSII reflect a lower quantum yield of PSII in drought-susceptible wheat, suggesting that drought-susceptible plants could not use absorbed irradiance efficiently via the photochemical reaction. In general, NPQ value increases when plants suffer from various environmental stresses^11,51^. The high level of NPQ in drought-susceptible wheat showed the increased need of dissipating excess light energy^31^. Furthermore, observations from the ultrastructure of thylakoid showed that osmotic stress changes the degree of grana stacking and destroys the intact structure of thylakoid (Fig. 9), thereby resulting in a decline in PSII activity in drought-susceptible wheat. It is well also demonstrated that the generation of different ROS in PSII is of importance when the electron transport chain between the photosystems is inhibited or the transfer of excitation energy is limited under environmental stresses^52^. However, excessive amounts of ROS could cause photoinhibition and oxidative damage, which will increase the susceptibility of PSII or even the whole photosynthetic apparatus to environmental stresses^13^. In the present study, the most robust changes in PSII activity and the chloroplast structure were showed in drought-susceptible wheat. Therefore, these results suggest that drought-resistant plants develop more effective protective mechanisms in avoiding PSII damages under environmental stresses.

Previous studies have indicated that osmotic stress adversely affects the levels of thylakoid membrane proteins^10,11,29^. In this study, we found that osmotic stress markedly decreased the amount of D1 in drought-susceptible wheat relative to drought-resistant wheat. The reason might be that osmotic stress resulted in the severe oxidative stress in drought-susceptible wheat, and subsequently down-regulated the rates of *PsbA* gene transcription and translation^10,27^. The significant decrease in the level of D1 protein may owe to the oxidative damage and photoinhibition in drought-susceptible wheat. In addition, a high level of phosphorylated Lhcb4 was found in drought-resistant wheat exposed to osmotic stress, indicating the key role of CP29 phosphorylation.

It has been reported that reversible phosphorylation of PSII proteins plays an important role in response of plants to environmental stresses^53,54^. However, the roles of reversible phosphorylation of PSII proteins and the assembly of thylakoid complexes between different wheat cultivars in response to water stress are not unknown. In this study, we found that osmotic stress caused a higher accumulation of PSII protein phosphorylation. In addition, our previous studies have indicated that reversible phosphorylation of PSII proteins is involved in plant resistance to environmental stresses in monocots^28,30^. In the present experiment, our results showed that drought-resistant wheat presented higher phosphorylated levels and higher dephosphorylated rate of PSII proteins under osmotic stress in comparison to drought-susceptible wheat. Rapid PSII protein dephosphorylation might be a regulatory response to environmental stresses in plant photosynthetic apparatus^29,30^. In addition, ROS production is a natural result of oxygenic photosynthesis. Thylakoid membrane protein complexes are especially susceptible to oxidative damage caused by ROS under environmental stresses. Our recent studies indicated that partial dissociation of LHCIIs from PSII and disassembly of LHCII assemblies occurred in different plants under the stressful conditions^11,55^. In the present study, the rapid disassembly of PSII-LHCII supercomplexes and LCHII assembly were observed in drought-susceptible wheat, suggesting that LHCII assemblies and the PSII-LHCII super-complex structural changes play a key role in stress resistance in plants.

In summary, the seedlings of drought-susceptible and drought-resistant wheat cultivars differed in their antioxidant enzymatic systems, ROS accumulation levels and photosynthetic characteristics. In the present study, the differential responses of two wheat cultivars to osmotic stress were shown. Our results indicated that drought-resistant wheat cultivar is more effective in preventing the oxidative damages and represents a high photosynthetic capacity. Moreover, the stronger phosphorylation and the rapid dephosphorylation of thylakoid membrane proteins were also observed in the drought-resistant wheat cultivar. Rapid disassembly of LHCII assemblies and the PSII-LHCII supercomplexes was found in the drought-susceptible wheat cultivar. In short, this study demonstrates that reversible phosphorylation of PSII proteins and assembly of thylakoid membrane protein complexes should be involved in plant resistance to osmotic stress.

## Methods

### Plant growth and stress treatment

Seedlings of two wheat (*Triticuma estivum* L.) cultivars, Chuanmai42, which is less sensitive to drought, and Sy95-71, which is more sensitive to drought^56^, were used in the experiments. Seeds were sterilized with 5% (w/v) sodium hypochlorite (NaClO) for 10 min, thoroughly rinsed with distilled water, placed in Petri dishes with wetted filter paper, and germinated in the dark at 25 °C in a greenhouse. The uniformly germinated seeds were planted in sterilized sand and grown in 1/2 Hoagland solution at 25 ± 1 °C under a 12 h photoperiod, with relative humidity 70%, photosynthetic photon flux density of 250 μmol m^−2^ s^−1^. For the osmotic stress treatment, seedlings form 2-week-old plants were carefully removed from the quartz sand, washed with tap water, and dried briefly with filter paper to remove surface water. Osmotic stress was conducted by submerging the roots of seedlings into half-strength Hoagland medium containing 20% (w/v) PEG-6000 with an osmotic potential of −0.53 MPa in beakers^28^. Control seedlings were cultured with 1/2 Hoagland solution and all samples were treated for 0, 24, and 72 h in the growth chamber.

For the antioxidant treatment, 5 mM ascorbid acid (AsA) was sprayed to the whole seedlings after osmotic stress for 0, 24, and 72 h in the growth chamber. 5 mM AsA also was applied in the 20% PEG-6000 during the osmotic stress.

### Determinations of chlorophyll (Chl), total protein content, relative water content (RWC), and osmotic regulators

Chlorophyll content was determined as the method described previously^57^. Seedling leaves (0.5 g fresh weight) were ground in 80:20 acetone: water mixture (v/v) at 25 °C. After filtering, absorbance of the filtrate was recorded at 663 and 645 nm. RWC was determined from fresh weight (FW), fresh weight at full turgor (TW) and dry weight (DW) of leaf [RWC = (FW − DW)/(TW − DW) × 100%]^58^. A UV spectrophotometer (Hitachi, Tokyo, Japan) was used for the measurement of total soluble proteins^59^. Soluble sugar was extracted in boiling water and its content was determined according to the method of Thomas^60^. Proline was extracted in 3% (w/v) sulfosalicylic acid. After filtration via filter paper, the solution at the absorbance of 520 nm was read by adding 0.75 mL ninhydrin reagent and glacial acetic-acid to the filtrate in boiled water for 1 h^61^.

### Qualitative and quantitative measurements of reactive oxygen species (ROS)

Leaf accumulations of superoxide anion (O_2_^.−^) and hydrogen peroxide (H_2_O_2_) were visually observed with nitro blue tetrazolium (NBT) and 3,3-diaminobenzidine (DAB), respectively, as described previously^32^. Detached leaf segments were immersed in 5 mM DAB for 8 h and 6 mM NBT for 2 h to detect H_2_O_2_ and O_2_^.−^, respectively. Then, de-colorization of leaf segments were performed in 95% ethanol (boiled at 90 °C for 0.5-2 h). The rate of O_2_^.−^ generation was measured according to a previously described method by Elstner and Heupel^62^ by monitoring the inhibition of the photochemical reduction of NBT. H_2_O_2_ content of leaves was colorimetrically analyzed following the method of Velikova *et al*.^63^ recording to the spectrum absorbance of the titanium-hydroperoxide complex.

### Electrolyte leakage and malonaldehyde content measurements

Electrolyte leakage (EL) was measured as described previously^32^. The calculation of the relative EL was done by the initial conductivity dividing the absolute conductivity, which was obtained by incubation in a boiling water bath for 15 min). The malonaldehyde (MDA) level was estimated based on the thiobarbituric acid reactive substance (TBARS) according to the method of Chen *et al*.^32^.

### Determination of antioxidant enzyme activities

Seedlings (0.3 g fresh weight) were homogenized on ice using extraction buffer (3 mL) that contained 50 mM sodium phosphate buffer (pH 7.8), 2% (w/v) polyvinylpyrrolidone (PVP) and 1 mM EDTA for the antioxidative-enzyme activity measurements. To measure the activity of ascorbate peroxidase (APX), 0.5 mM ascorbate was added in the extraction buffer. To determine the activities of dehydroascorbate reductase (DHAR) and glutathione reductase (GR), the extraction buffer contained 2 mM *β*-mercaptoethanol. After 12,000 *g* centrifugation (30 min at 4 °C), the resulting supernatant was collected for the enzyme assays. Superoxide dismutase (SOD) activity was assayed by recording the inhibition of cytochrome c reduction by xanthine oxidase^64^. One unit of SOD was defined as the amount of enzyme required to inhibit the reduction of cytochrome c by 50%. Catalase (CAT) activity was done by determining the initial rate of disappearance of H_2_O_2_ at 240 nm (extinction coefficient of 39.4 mM^−1^ cm^−1^)^65^. The reaction mixture in a total volume of 3 mL contained 50 mM phosphate buffer (pH 7.0), 50 μL enzyme, and 12.5 mM H_2_O_2_. Peroxidase (POD) activity was estimated based on the increase in the absorbance at 470 nm as the formation of the guaiacol oxidation product (extinction coefficient of 26.6 mM^−1^ cm^−1^)^66^. The reaction mixture in a total volume of 3 mL contained 50 mM sodium phosphate buffer (pH 7.0), 0.05% guaiacol, 0.1 mM EDTA, 50 μL enzyme, and 1 mM H_2_O_2_. The APX activity was measured by monitoring the decrease in the content of ascorbate (AsA) at 290 nm (extinction coefficient of 2.8 mM^−1^ cm^−1^)^65^. The reaction mixture in a total volume of 3 mL contained 50 mM (pH 7.0) phosphate buffer, 0.1 mM EDTA, 0.5 mM ascorbic acid, 100 μL of enzyme extract, and 150 mM H_2_O_2_. DHAR was measured through reading the formation of AsA at 265 nm (extinction coefficient of 14 mM^−1^ cm^−1^)^66^. The reaction mixture in a total volume of 3 mL consisted of 50 mM (pH 7.0) sodium phosphate buffer, 3.5 mM GSH, 0.1 mM EDTA, 100 μL enzyme extract, and 0.4 mM DHAR. The GR activity was determined based on the decline in the absorbance at 340 nm (extinction coefficient of 6.2 mM^−1^ cm^−1^) because of NADPH oxidation^66^. The reaction mixture in a total of 3 mL contained 50 mM (pH 7.0) sodium phosphate buffer, 0.5 mM oxidized glutathione (GSSG), 0.1 mM EDTA, 100 μL enzyme extract, and 0.12 mM NADPH.

Measurements of dehydroascorbate and reduced ascorbic acid (DHA and AsA) in 5% (w/v) of trichloroacetic acid were performed according to Cakmak and Marschner^67^. 0.5 g fresh leaves were frozen and ground to a fine powder in liquid nitrogen. AsA was extracted in ice-cold 5% (w/v) metaphosphoric acid, and then 15 min centrifugation (22,000 *g* at 4 °C) was performed. To measure the concentration of total ascorbate (AsA + DHA), the reaction mixture contained 0.2 mL supernatant, 150 mM phosphate buffer (0.5 mL, pH 7.5) with 5 mM EDTA, and 10 mM DTT (0.1 mL). To measure the content of AsA, 0.2 mL supernatant, 150 mM phosphate buffer (0.5 mL, pH 7.5), and 0.2 mL water were contained in the reaction mixture. The assay is conducted according to the reduction of Fe^3+^ to Fe^2+^ by AsA. Fe^2+^ can bind with bipyridyl, and then gives a pink color. The color produced was recorded at 525 nm. Total ascorbate was determined after reduction of DHA to AsA with DTT. Then, DHA concentration was estimated by the difference between total ascorbate and AsA.

### Trypan-blue staining

Tissue staining with trypan blue (1.25 mg ml^−1^, sigma) was carried out according to the method of Koch and Slusarenko^68^. The second leaves after different treatments were used for trypan-blue staining. After staining, the leaves were cleared by 2.5 g mL^−1^ chloral hydrate solution.

### Measurements of Chlorophyll fluorescence and gas exchange

An imaging PAM M-Series fluorometer (Heinz-Walz Instruments, Effeltrich, Germany) was applied in measuring chlorophyll fluorescence following the manufacturer’s instructions at room temperature. A saturating light intensity of 8000 µmol photons m^−2^ s^−1^ and white actinic light of 1500 µmol photons m^−2^ s^−1^ were used in the present experiment. Leaves were adapted for half an hour before measurement in the darkness. Minimum fluorescence (Fo) and maximum fluorescence (Fm) were determined using dark-adapted samples, from which the PSII maximum quantum yield (Fv/Fm) value derived. Fluorescence levels were calculated according to the following equations^69^: the photochemical quenching (qP) = (Fm′ − Fs)/(Fm′ − Fo′), the non-photochemical quenching (NPQ) = (Fm − Fm′)/Fm′, and the quantum yield of PSII electron transport (ΦPSII) = (Fm′ − Fs)/Fm′, where Fm′ is the maximum chlorophyll fluorescence under actinic light exposure, Fs the steady-state fluorescence during illumination, and Fo′ the minimal fluorescence during illumination.

Transpiration and stomatal conductance were measured using the GSF-3000 photosynthesis system (Heinz-Walz Instruments, Effeltrich, Germany) following the instructions provided by the manufacturer. In the assimilation chamber, external CO_2_ concentration was set to 360 µmol mol^−1^ at room temperature (25 °C), relative humidity in the range 60–80%, and light intensity was set to 600 µmol photons m^−2^ s^−1^.

### Immunoblotting analysis of thylakoid membrane proteins

Thylakoid proteins from wheat leaves were prepared based on the previous method with NaF^70^. Total chlorophyll concentrations of thylakoid preparation were measured according to the previous method^57^. Separation of the extracted thylakoid proteins were performed by SDS-PAGE on a 14% acrylamide^71^. Proteins were electro-blotted onto a polyvinylidene difluoride membrane (Immobilone, Millipore, Darmstadt, Germany). LHCa3, CP24, CP26, CP29, LHCb3, LHCb2, LHCb1, CP43, D2, and D1 were immuno-detected with protein-specific antibodies^70^. These specific antibodies in the present experiment were bought from Agrisera, Sweden. For protein phosphorylation assay, thylakoids were immuno-decorated with a polyclonal anti-phosphothreonine antibody from New England Biolabs (Cell Signalling, http://www.neb.com/)^72^. Then, a horseradish peroxidase-linked secondary antibody in conjunction with chem-iluminescent agent (GE Healthcare, www.gehealthcare.com) was adopted.

For dephosphorylation analyses, wheat plants were illuminated under a photosynthetic photon flux density of 1000 μmol photons m^−2^ s^−1^ to phosphorylate PSII reaction center proteins or at 80 μmol photons m^−2^ s^−1^ to induce maximal LHCII phosphorylation for 60 min in the greenhouse^29^. After light treatment, wheat plants were transferred to darkness and adapted for 120 min to dephosphorylation gradually in the growth chamber. Wheat samples for extracting thylakoid membranes were prepared during the time course of incubation, frozen in liquid nitrogen, and stored at −80 °C. Signal amplitude of the immunoblots was quantified through Quantity One software (Bio-Rad Comp. Hercules, CA, USA).

### Blue-native electrophoresis

Blue-native-PAGE (BN-PAGE) was carried out following described previously^73^ with slight modifications. Thylakoid membrane samples (20 μg of Chl) were solubilized in ice-cold resuspension buffer on ice for 10 min in the darkness in the presence of 1% (w/v) *n*-dodecyl-*β*-_D_-maltoside (DM) (Sigma-Aldrich, http://www.sigmaaldrich.com/). The electrophoresis was carried out at 4 °C until the sample reached the end of the gel. After BN-PAGE, the protein immunoblotting was carried out as described by Witting *et al*.^74^. Quantification of thylakoid membrane complexes was done using Quantity One software (Bio-Rad Comp. Hercules, CA, USA).

### Transmission electron microscopy analysis

Thylakoid ultrastructure was analyzed according to Liu *et al*.^29^. Wheat leaf samples from drought-resistant and drought-susceptible wheat were fixed with 3% glutaraldehyde in 0.1 M sodium cacodylate buffer (pH 6.9) overnight at 4 °C, then fixed with 1% osmium tetroxide, dehydrated with acetone and embedded in Epon 812. Thin sections were cut with an ultramicrotome (Ultracut F-701704, Reichert-Jung, Reichert, Austria) were negatively stained with 2% uranyl acetate on glow discharged carbon-coated copper grids. Electron microscopy was performed using a TEM H600 electron microscope Hitachi, Midland, ON, Canada) operating at 100 kV.

### Statistics analysis

The data analysis in the present experiment was conducted using the SPSS statistical 19.0 software (IBM, Chicago, IL, USA). All results were presented with mean ± standard deviations (SD). Each treatment value was at least in triplicate. Bars with different letters above the columns of figures indicated significant differences at *P* < 0.05 (Duncan’s multiplication range test).

## Electronic supplementary material

Supplementary information
