# Supplementary material for: Comparison of phosphorylation and assembly of photosystem complexes and redox homeostasis in two wheat cultivars with different drought resistance
Source: Sci Rep. 2017 Oct 5;7:12718. doi: 10.1038/s41598-017-13145-1 (PMC5629198; doi:10.1038/s41598-017-13145-1)

Comparison of phosphorylation and assembly of photosystem complexes and redox homeostasis in two wheat cultivars with different drought resistance

Yang-Er Chen[1,*], Jun-Mei Cui[1,*], Yan-Qiu Su[2,*], Chao-Ming Zhang[1], Jie Ma[1], Zhong-Wei Zhang[3], Ming Yuan[1], Wen-Juan Liu[4], Huai-Yu Zhang[1], Shu Yuan[3]

[1]College of Life Sciences, Sichuan Agricultural University, Ya'an 625014, China

[2]College of Life Science, Sichuan University, Chengdu 610064, China

[3]College of Resources, Sichuan Agricultural University, Chengdu 611130, China

[4]Center of Analysis and Testing, Sichuan Academy of Agricultural Sciences, Chengdu 610066, China

*These authors contribute equally to this work.

Correspondence and requests for materials should be addressed to Y. E. C. (e-mail: anty9826@163.com) or S. Y. (e-mail: roundtree318@hotmail.com)

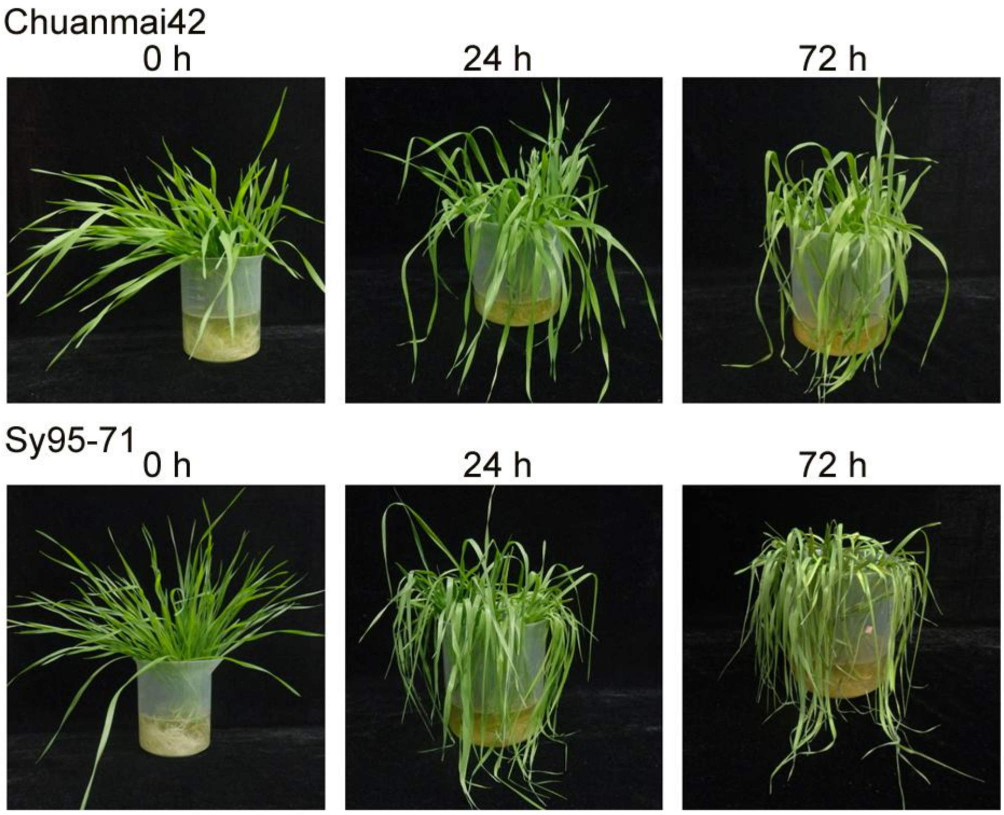

Fig. S1. Symptoms in Chuanmai42 and Sy95-71 under osmotic stress.0-72 h represents osmotic stress for 0 h (control), 24 h, and 72 h.

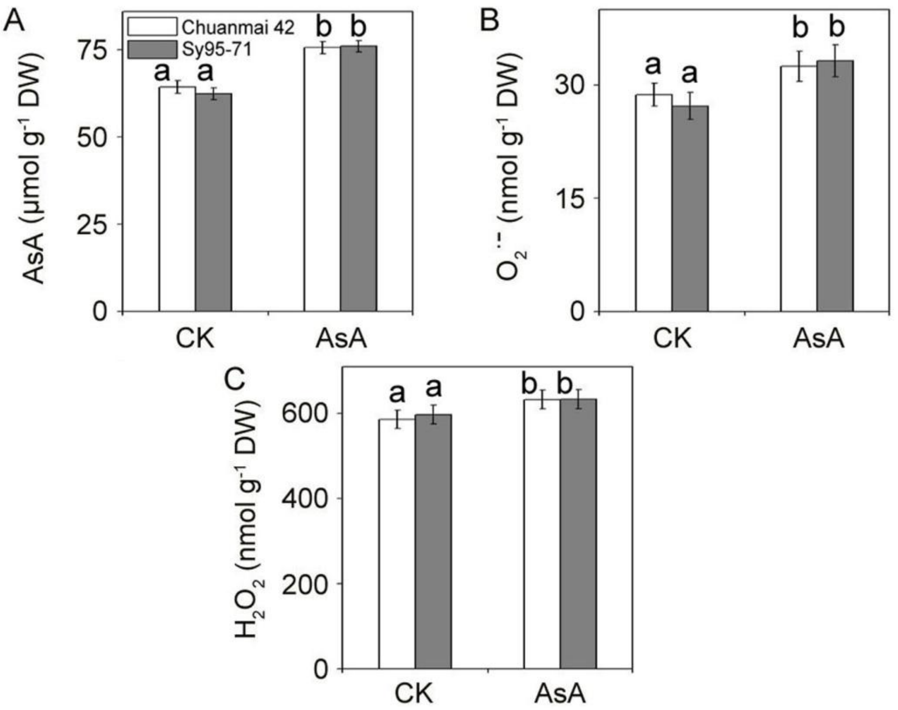

Fig. S2. Effects of exogenous AsA treatment on the content of AsA (A), superoxide anion radicals ($O_2^{\bullet-}$) production rate (B), and hydrogen peroxide (H_2O_2) content (C) in Chuanmai42 and Sy95-71 under non-stressful conditions. Bars represent standard deviations of three independent biological replicates (*n* = 3) and values followed by the same letter are not significantly different ($P > 0.05$). CK represents control seedlings. AsA represents 5 mM AsA treatment under non-osmotic stress.

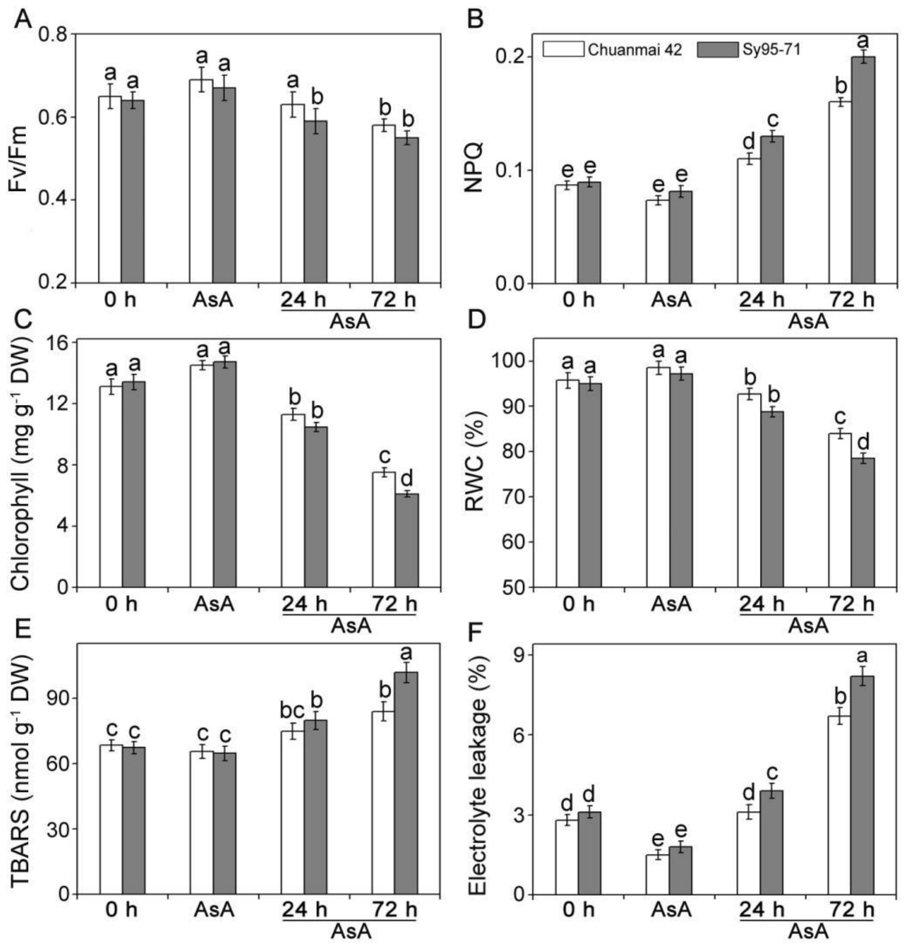

Fig. S3. Effects of AsA treatment on chlorophyll fluorescence parameters (Fv/Fm, the maximum efficiency of PSII photochemistry; NPQ/4, non-photochemical quenching coefficient) (A, B), chlorophyll (Chl) content (C), relative water content (RWC) (D), MDA (E), and electrolyte leakage (F) under osmotic stress in Chuanmai42 and Sy95-71. Bars represent standard deviations of three independent biological replicates (n = 3) and values followed by the same letter are not significantly different ($P > 0.05$). 0-72 h represents osmotic stress for 0 h (control), 24 h, and 72 h. AsA represents 5 mM AsA treatment under non-osmotic stress.

Fig. 7B

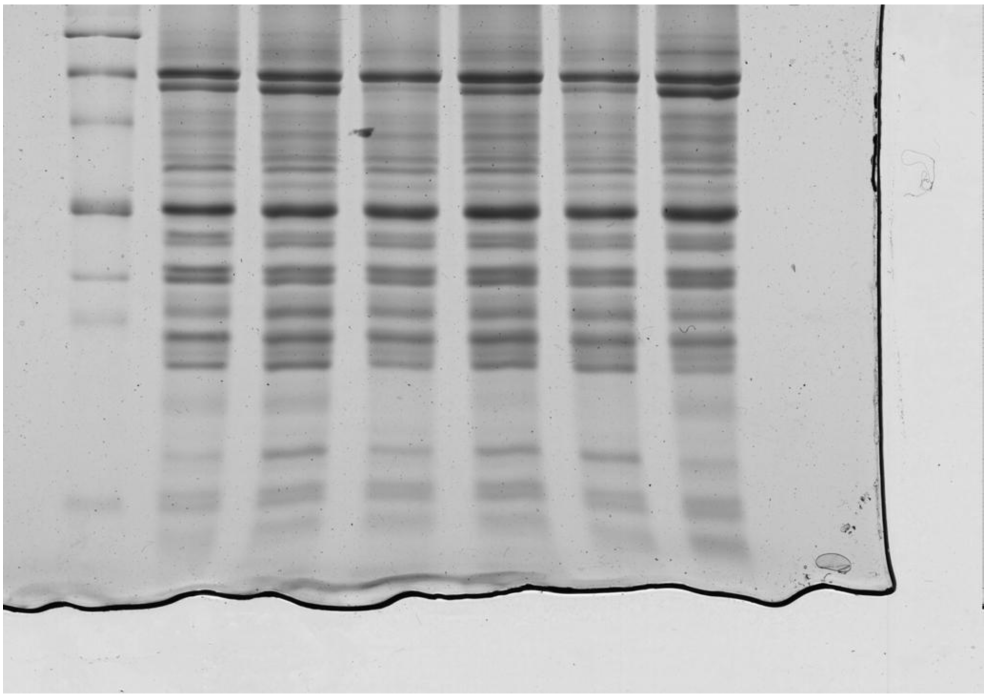

Fig. 9A

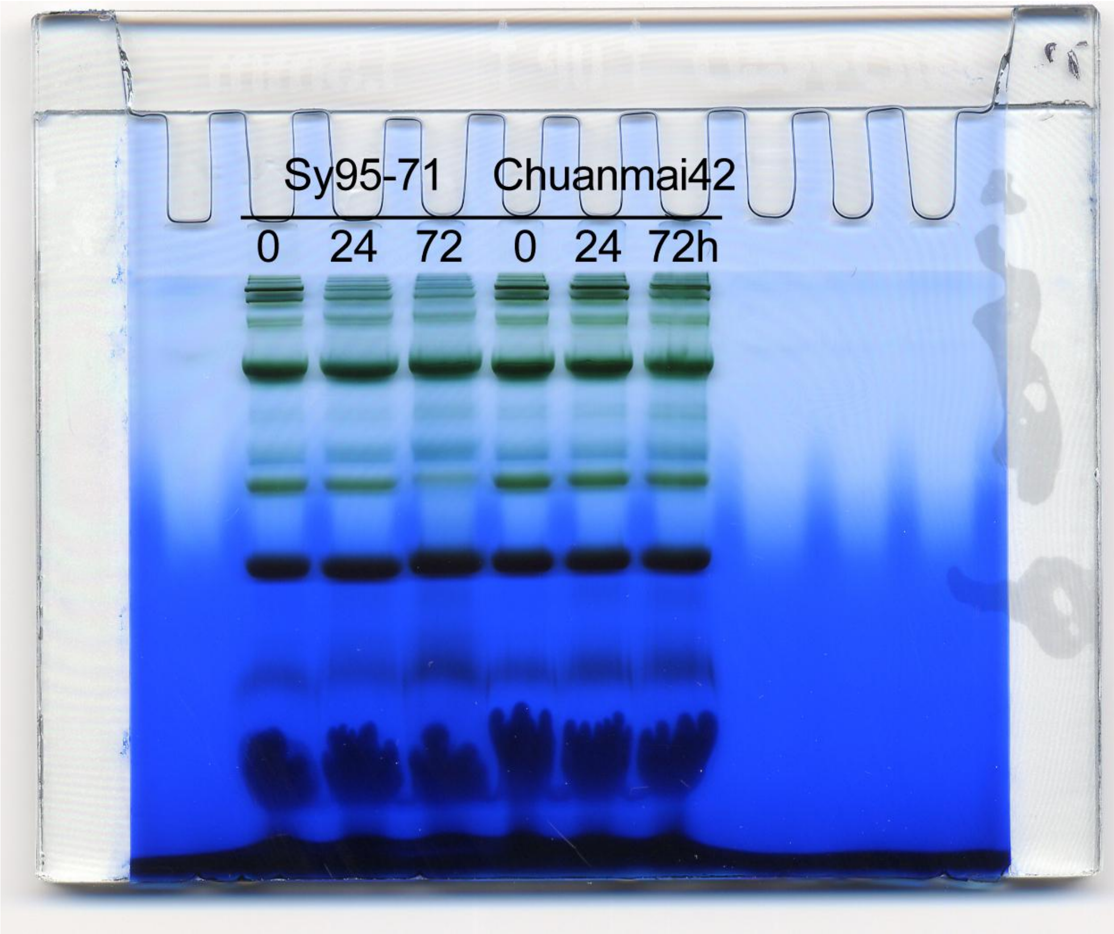

Supplement: Supplementary file 1 — Supplementary information [file 41598_2017_13145_MOESM1_ESM.pdf]
